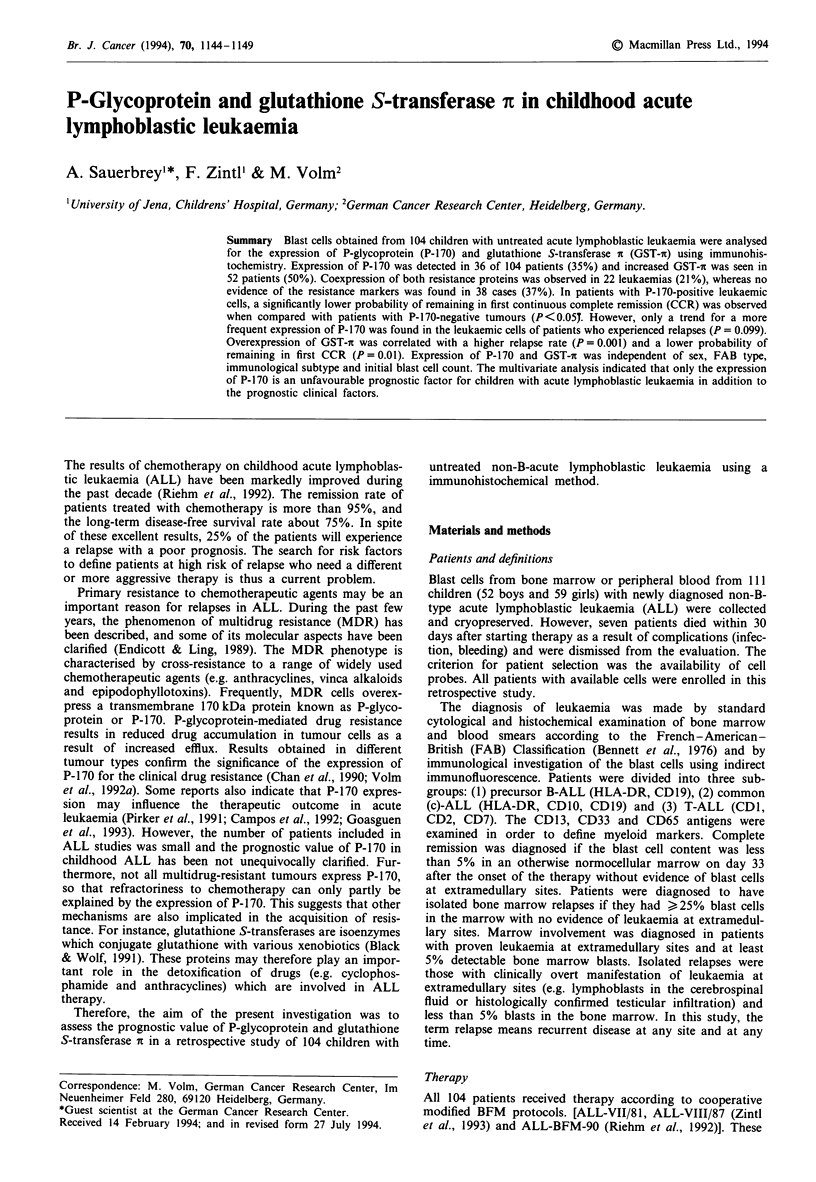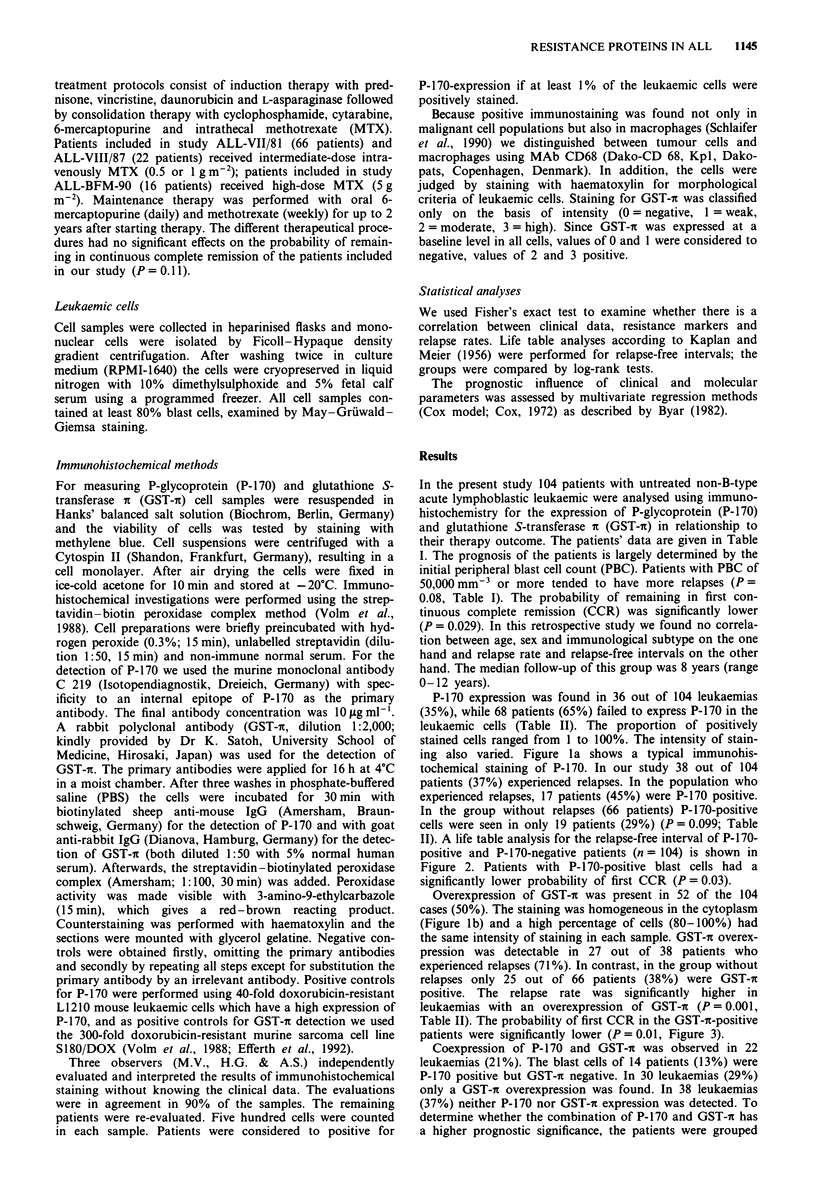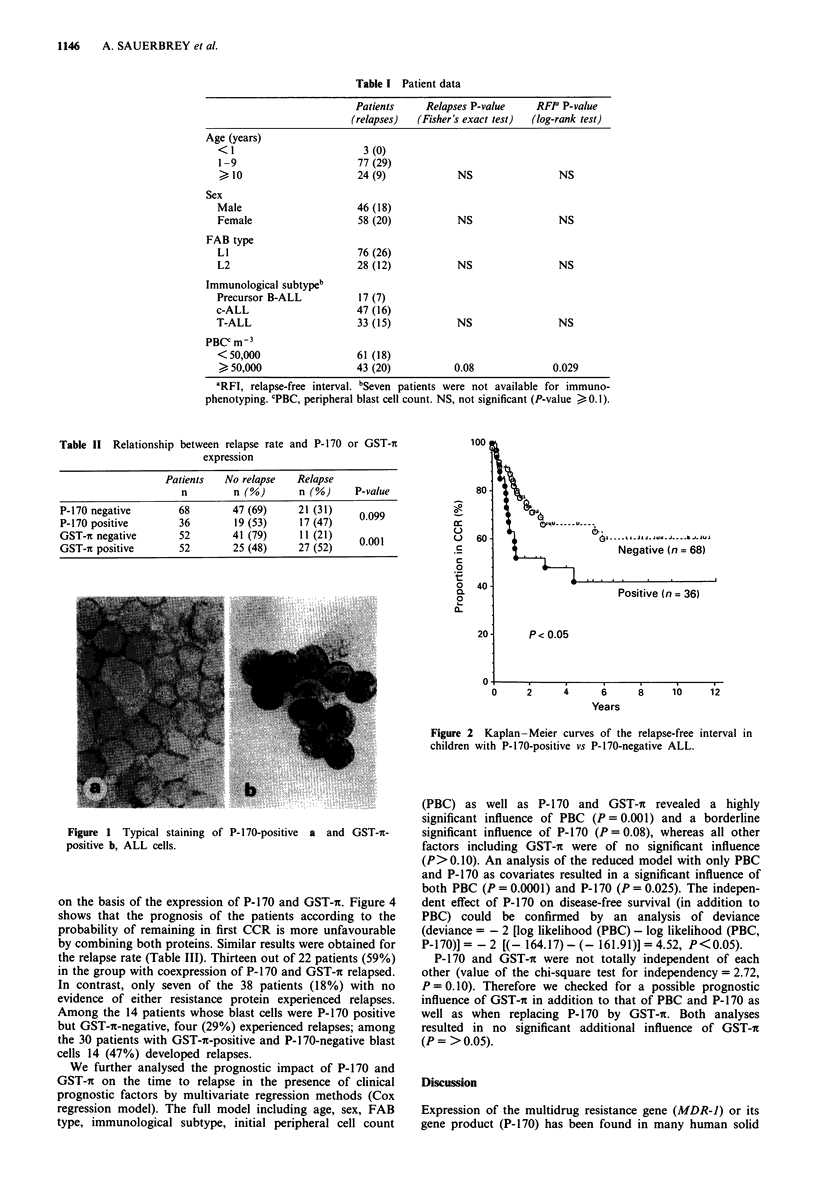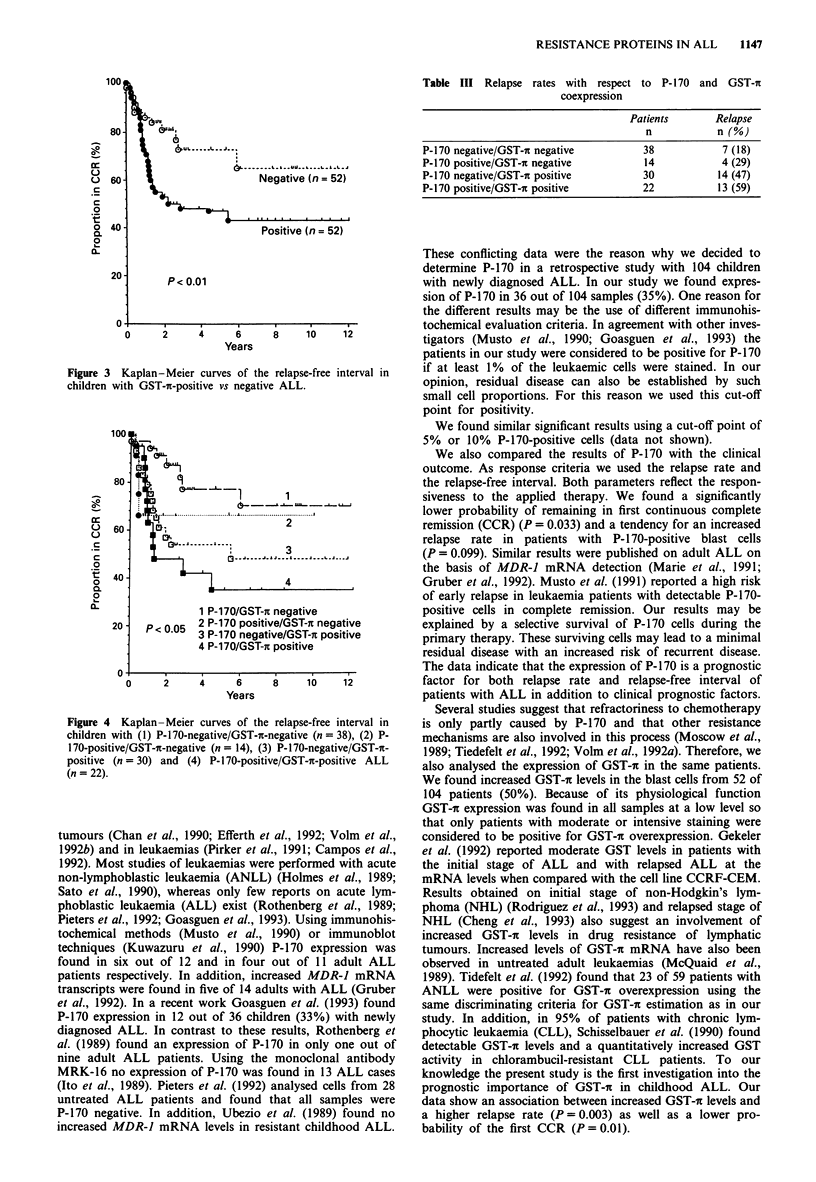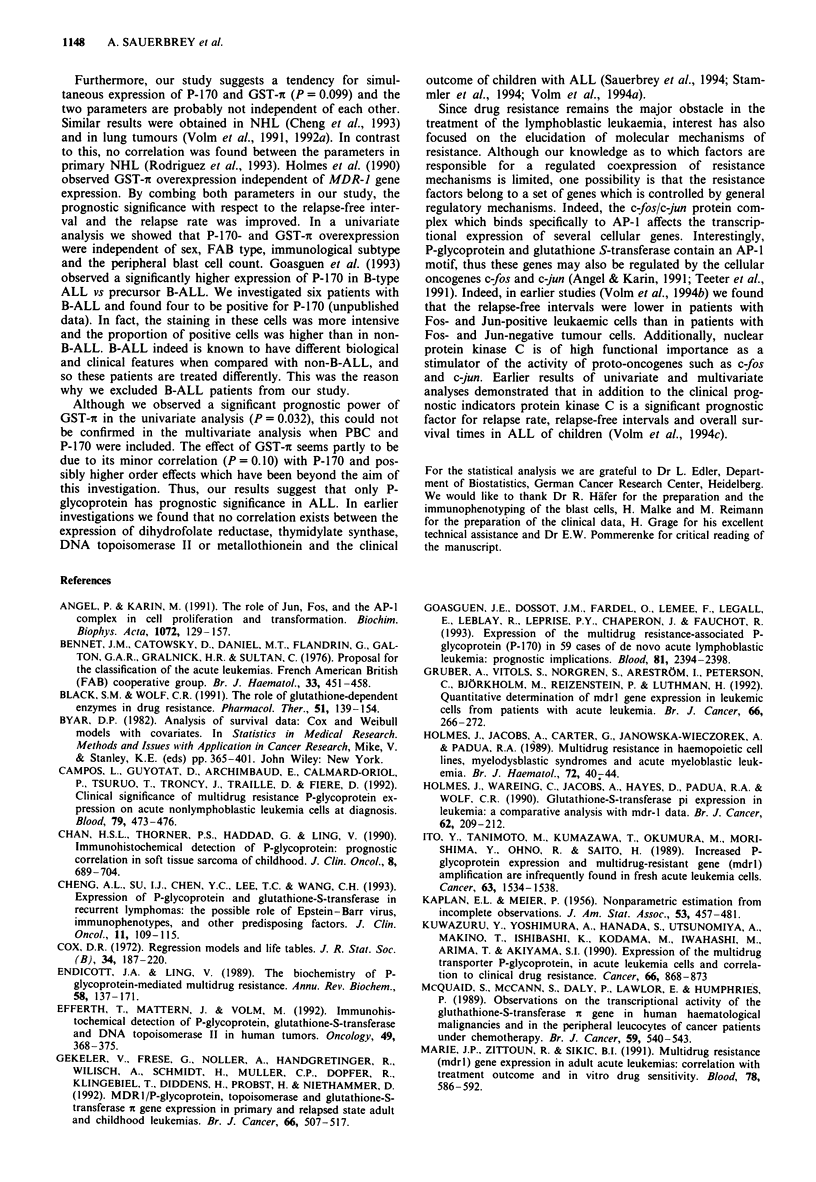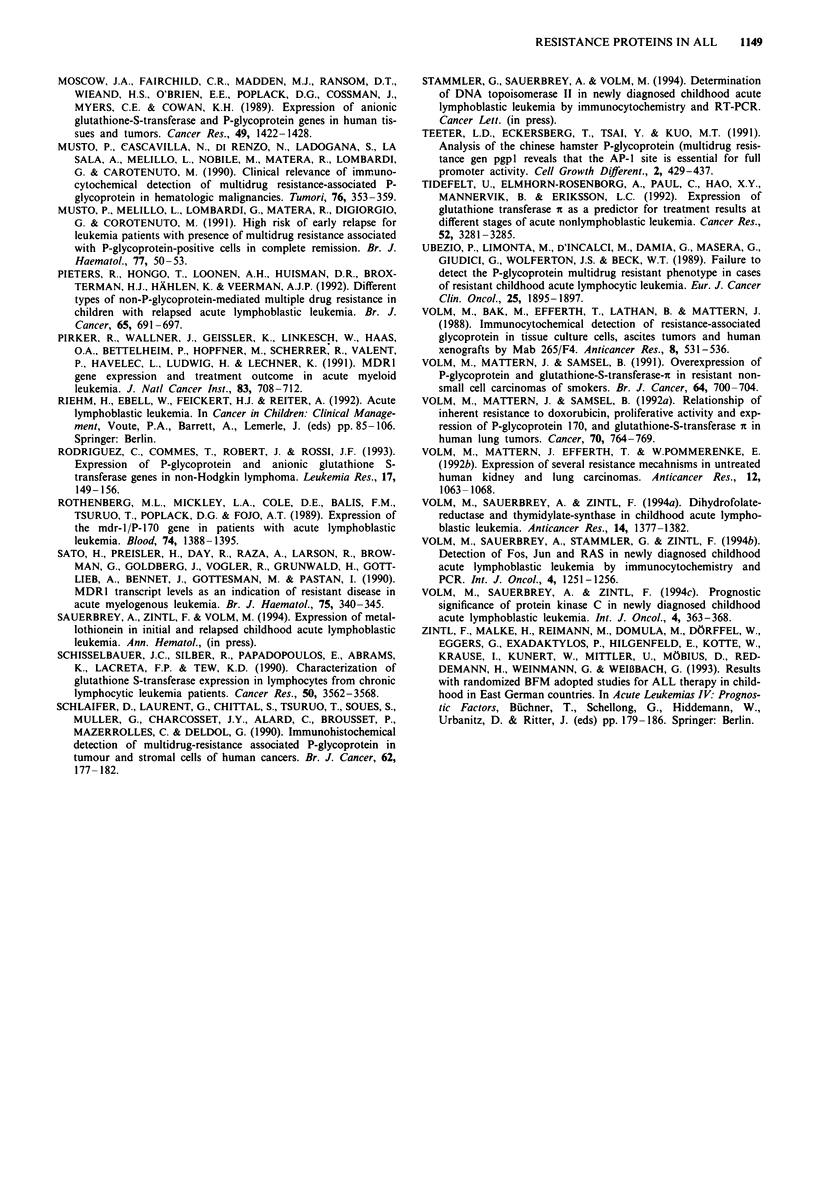# P-glycoprotein and glutathione S-transferase pi in childhood acute lymphoblastic leukaemia.

**DOI:** 10.1038/bjc.1994.462

**Published:** 1994-12

**Authors:** A. Sauerbrey, F. Zintl, M. Volm

**Affiliations:** University of Jena, Children's Hospital, Germany.

## Abstract

**Images:**


Br. J. Cancer (1994), 70, 1144-1149 © Macmillan Press Ltd., 1994

### P-Glycoprotein and glutathione *S*-transferase π in childhood acute lymphoblastic leukaemia

A. Sauerbrey[1]*, F. Zintl[1] & M. Volm[2]

[1]*University of Jena, Childrens' Hospital, Germany;* [2]*German Cancer Research Center, Heidelberg, Germany.*

**Summary** Blast cells obtained from 104 children with untreated acute lymphoblastic leukaemia were analysed for the expression of P-glycoprotein (P-170) and glutathione *S*-transferase π (GST-π) using immunohistochemistry. Expression of P-170 was detected in 36 of 104 patients (35%) and increased GST-π was seen in 52 patients (50%). Coexpression of both resistance proteins was observed in 22 leukaemias (21%), whereas no evidence of the resistance markers was found in 38 cases (37%). In patients with P-170-positive leukaemic cells, a significantly lower probability of remaining in first continuous complete remission (CCR) was observed when compared with patients with P-170-negative tumours ($P<0.05$). However, only a trend for a more frequent expression of P-170 was found in the leukaemic cells of patients who experienced relapses ($P = 0.099$). Overexpression of GST-π was correlated with a higher relapse rate ($P = 0.001$) and a lower probability of remaining in first CCR ($P = 0.01$). Expression of P-170 and GST-π was independent of sex, FAB type, immunological subtype and initial blast cell count. The multivariate analysis indicated that only the expression of P-170 is an unfavourable prognostic factor for children with acute lymphoblastic leukaemia in addition to the prognostic clinical factors.

The results of chemotherapy on childhood acute lymphoblastic leukaemia (ALL) have been markedly improved during the past decade (Riehm *et al.*, 1992). The remission rate of patients treated with chemotherapy is more than 95%, and the long-term disease-free survival rate about 75%. In spite of these excellent results, 25% of the patients will experience a relapse with a poor prognosis. The search for risk factors to define patients at high risk of relapse who need a different or more aggressive therapy is thus a current problem.

Primary resistance to chemotherapeutic agents may be an important reason for relapses in ALL. During the past few years, the phenomenon of multidrug resistance (MDR) has been described, and some of its molecular aspects have been clarified (Endicott & Ling, 1989). The MDR phenotype is characterised by cross-resistance to a range of widely used chemotherapeutic agents (e.g. anthracyclines, vinca alkaloids and epipodophyllotoxins). Frequently, MDR cells overexpress a transmembrane 170 kDa protein known as P-glycoprotein or P-170. P-glycoprotein-mediated drug resistance results in reduced drug accumulation in tumour cells as a result of increased efflux. Results obtained in different tumour types confirm the significance of the expression of P-170 for the clinical drug resistance (Chan *et al.*, 1990; Volm *et al.*, 1992*a*). Some reports also indicate that P-170 expression may influence the therapeutic outcome in acute leukaemia (Pirker *et al.*, 1991; Campos *et al.*, 1992; Goasguen *et al.*, 1993). However, the number of patients included in ALL studies was small and the prognostic value of P-170 in childhood ALL has been not unequivocally clarified. Furthermore, not all multidrug-resistant tumours express P-170, so that refractoriness to chemotherapy can only partly be explained by the expression of P-170. This suggests that other mechanisms are also implicated in the acquisition of resistance. For instance, glutathione *S*-transferases are isoenzymes which conjugate glutathione with various xenobiotics (Black & Wolf, 1991). These proteins may therefore play an important role in the detoxification of drugs (e.g. cyclophosphamide and anthracyclines) which are involved in ALL therapy.

Therefore, the aim of the present investigation was to assess the prognostic value of P-glycoprotein and glutathione *S*-transferase π in a retrospective study of 104 children with untreated non-B-acute lymphoblastic leukaemia using a immunohistochemical method.

Correspondence: M. Volm, German Cancer Research Center, Im Neuenheimer Feld 280, 69120 Heidelberg, Germany.
*Guest scientist at the German Cancer Research Center.
Received 14 February 1994; and in revised form 27 July 1994.

### Materials and methods

### *Patients and definitions*

Blast cells from bone marrow or peripheral blood from 111 children (52 boys and 59 girls) with newly diagnosed non-B-type acute lymphoblastic leukaemia (ALL) were collected and cryopreserved. However, seven patients died within 30 days after starting therapy as a result of complications (infection, bleeding) and were dismissed from the evaluation. The criterion for patient selection was the availability of cell probes. All patients with available cells were enrolled in this retrospective study.

The diagnosis of leukaemia was made by standard cytological and histochemical examination of bone marrow and blood smears according to the French-American-British (FAB) Classification (Bennett *et al.*, 1976) and by immunological investigation of the blast cells using indirect immunofluorescence. Patients were divided into three subgroups: (1) precursor B-ALL (HLA-DR, CD19), (2) common (c)-ALL (HLA-DR, CD10, CD19) and (3) T-ALL (CD1, CD2, CD7). The CD13, CD33 and CD65 antigens were examined in order to define myeloid markers. Complete remission was diagnosed if the blast cell content was less than 5% in an otherwise normocellular marrow on day 33 after the onset of the therapy without evidence of blast cells at extramedullary sites. Patients were diagnosed to have isolated bone marrow relapses if they had ⩾25% blast cells in the marrow with no evidence of leukaemia at extramedullary sites. Marrow involvement was diagnosed in patients with proven leukaemia at extramedullary sites and at least 5% detectable bone marrow blasts. Isolated relapses were those with clinically overt manifestation of leukaemia at extramedullary sites (e.g. lymphoblasts in the cerebrospinal fluid or histologically confirmed testicular infiltration) and less than 5% blasts in the bone marrow. In this study, the term relapse means recurrent disease at any site and at any time.

### *Therapy*

All 104 patients received therapy according to cooperative modified BFM protocols. [ALL-VII/81, ALL-VIII/87 (Zintl *et al.*, 1993) and ALL-BFM-90 (Riehm *et al.*, 1992)]. These treatment protocols consist of induction therapy with prednisone, vincristine, daunorubicin and L-asparaginase followed by consolidation therapy with cyclophosphamide, cytarabine, 6-mercaptopurine and intrathecal methotrexate (MTX). Patients included in study ALL-VII/81 (66 patients) and ALL-VIII/87 (22 patients) received intermediate-dose intravenously MTX (0.5 or 1 g $m^{-2}$); patients included in study ALL-BFM-90 (16 patients) received high-dose MTX (5 g $m^{-2}$). Maintenance therapy was performed with oral 6-mercaptopurine (daily) and methotrexate (weekly) for up to 2 years after starting therapy. The different therapeutical procedures had no significant effects on the probability of remaining in continuous complete remission of the patients included in our study ($P = 0.11$).

*Leukaemic cells*

Cell samples were collected in heparinised flasks and mononuclear cells were isolated by Ficoll–Hypaque density gradient centrifugation. After washing twice in culture medium (RPMI-1640) the cells were cryopreserved in liquid nitrogen with 10% dimethylsulphoxide and 5% fetal calf serum using a programmed freezer. All cell samples contained at least 80% blast cells, examined by May–Grüwald–Giemsa staining.

*Immunohistochemical methods*

For measuring P-glycoprotein (P-170) and glutathione *S*-transferase π (GST-π) cell samples were resuspended in Hanks' balanced salt solution (Biochrom, Berlin, Germany) and the viability of cells was tested by staining with methylene blue. Cell suspensions were centrifuged with a Cytospin II (Shandon, Frankfurt, Germany), resulting in a cell monolayer. After air drying the cells were fixed in ice-cold acetone for 10 min and stored at −20°C. Immunohistochemical investigations were performed using the streptavidin–biotin peroxidase complex method (Volm *et al.*, 1988). Cell preparations were briefly preincubated with hydrogen peroxide (0.3%; 15 min), unlabelled streptavidin (dilution 1:50, 15 min) and non-immune normal serum. For the detection of P-170 we used the murine monoclonal antibody C 219 (Isotopendiagnostik, Dreieich, Germany) with specificity to an internal epitope of P-170 as the primary antibody. The final antibody concentration was 10 μg $ml^{-1}$. A rabbit polyclonal antibody (GST-π, dilution 1:2,000; kindly provided by Dr K. Satoh, University School of Medicine, Hirosaki, Japan) was used for the detection of GST-π. The primary antibodies were applied for 16 h at 4°C in a moist chamber. After three washes in phosphate-buffered saline (PBS) the cells were incubated for 30 min with biotinylated sheep anti-mouse IgG (Amersham, Braunschweig, Germany) for the detection of P-170 and with goat anti-rabbit IgG (Dianova, Hamburg, Germany) for the detection of GST-π (both diluted 1:50 with 5% normal human serum). Afterwards, the streptavidin–biotinylated peroxidase complex (Amersham; 1:100, 30 min) was added. Peroxidase activity was made visible with 3-amino-9-ethylcarbazole (15 min), which gives a red–brown reacting product. Counterstaining was performed with haematoxylin and the sections were mounted with glycerol gelatine. Negative controls were obtained firstly, omitting the primary antibodies and secondly by repeating all steps except for substitution the primary antibody by an irrelevant antibody. Positive controls for P-170 were performed using 40-fold doxorubicin-resistant L1210 mouse leukaemic cells which have a high expression of P-170, and as positive controls for GST-π detection we used the 300-fold doxorubicin-resistant murine sarcoma cell line S180/DOX (Volm *et al.*, 1988; Efferth *et al.*, 1992).

Three observers (M.V., H.G. & A.S.) independently evaluated and interpreted the results of immunohistochemical staining without knowing the clinical data. The evaluations were in agreement in 90% of the samples. The remaining patients were re-evaluated. Five hundred cells were counted in each sample. Patients were considered to positive for P-170-expression if at least 1% of the leukaemic cells were positively stained.

Because positive immunostaining was found not only in malignant cell populations but also in macrophages (Schlaifer *et al.*, 1990) we distinguished between tumour cells and macrophages using MAb CD68 (Dako-CD 68, Kpl, Dakopats, Copenhagen, Denmark). In addition, the cells were judged by staining with haematoxylin for morphological criteria of leukaemic cells. Staining for GST-π was classified only on the basis of intensity (0 = negative, 1 = weak, 2 = moderate, 3 = high). Since GST-π was expressed at a baseline level in all cells, values of 0 and 1 were considered to negative, values of 2 and 3 positive.

*Statistical analyses*

We used Fisher's exact test to examine whether there is a correlation between clinical data, resistance markers and relapse rates. Life table analyses according to Kaplan and Meier (1956) were performed for relapse-free intervals; the groups were compared by log-rank tests.

The prognostic influence of clinical and molecular parameters was assessed by multivariate regression methods (Cox model; Cox, 1972) as described by Byar (1982).

### Results

In the present study 104 patients with untreated non-B-type acute lymphoblastic leukaemic were analysed using immunohistochemistry for the expression of P-glycoprotein (P-170) and glutathione *S*-transferase π (GST-π) in relationship to their therapy outcome. The patients' data are given in Table I. The prognosis of the patients is largely determined by the initial peripheral blast cell count (PBC). Patients with PBC of 50,000 $mm^{-3}$ or more tended to have more relapses ($P = 0.08$, Table I). The probability of remaining in first continuous complete remission (CCR) was significantly lower ($P = 0.029$). In this retrospective study we found no correlation between age, sex and immunological subtype on the one hand and relapse rate and relapse-free intervals on the other hand. The median follow-up of this group was 8 years (range 0–12 years).

P-170 expression was found in 36 out of 104 leukaemias (35%), while 68 patients (65%) failed to express P-170 in the leukaemic cells (Table II). The proportion of positively stained cells ranged from 1 to 100%. The intensity of staining also varied. Figure 1a shows a typical immunohistochemical staining of P-170. In our study 38 out of 104 patients (37%) experienced relapses. In the population who experienced relapses, 17 patients (45%) were P-170 positive. In the group without relapses (66 patients) P-170-positive cells were seen in only 19 patients (29%) ($P = 0.099$; Table II). A life table analysis for the relapse-free interval of P-170-positive and P-170-negative patients ($n = 104$) is shown in Figure 2. Patients with P-170-positive blast cells had a significantly lower probability of first CCR ($P = 0.03$).

Overexpression of GST-π was present in 52 of the 104 cases (50%). The staining was homogeneous in the cytoplasm (Figure 1b) and a high percentage of cells (80–100%) had the same intensity of staining in each sample. GST-π overexpression was detectable in 27 out of 38 patients who experienced relapses (71%). In contrast, in the group without relapses only 25 out of 66 patients (38%) were GST-π positive. The relapse rate was significantly higher in leukaemias with an overexpression of GST-π ($P = 0.001$, Table II). The probability of first CCR in the GST-π-positive patients were significantly lower ($P = 0.01$, Figure 3).

Coexpression of P-170 and GST-π was observed in 22 leukaemias (21%). The blast cells of 14 patients (13%) were P-170 positive but GST-π negative. In 30 leukaemias (29%) only a GST-π overexpression was found. In 38 leukaemias (37%) neither P-170 nor GST-π expression was detected. To determine whether the combination of P-170 and GST-π has a higher prognostic significance, the patients were grouped

**Table I** Patient data

| | Patients (relapses) | Relapses P-value (Fisher's exact test) | RFI[a] P-value (log-rank test) |
|---|---|---|---|
| Age (years) | | | |
| <1 | 3 (0) | | |
| 1–9 | 77 (29) | | |
| ≥10 | 24 (9) | NS | NS |
| Sex | | | |
| Male | 46 (18) | | |
| Female | 58 (20) | NS | NS |
| FAB type | | | |
| L1 | 76 (26) | | |
| L2 | 28 (12) | NS | NS |
| Immunological subtype[b] | | | |
| Precursor B-ALL | 17 (7) | | |
| c-ALL | 47 (16) | | |
| T-ALL | 33 (15) | NS | NS |
| PBC[c] $m^{-3}$ | | | |
| <50,000 | 61 (18) | | |
| ≥50,000 | 43 (20) | 0.08 | 0.029 |

[a]RFI, relapse-free interval. [b]Seven patients were not available for immunophenotyping. [c]PBC, peripheral blast cell count. NS, not significant (*P*-value ≥0.1).

**Table II** Relationship between relapse rate and P-170 or GST-π expression

| | Patients n | No relapse n (%) | Relapse n (%) | P-value |
|---|---|---|---|---|
| P-170 negative | 68 | 47 (69) | 21 (31) | 0.099 |
| P-170 positive | 36 | 19 (53) | 17 (47) | |
| GST-π negative | 52 | 41 (79) | 11 (21) | 0.001 |
| GST-π positive | 52 | 25 (48) | 27 (52) | |

**Figure 1** Typical staining of P-170-positive **a** and GST-π-positive **b**, ALL cells.

**Figure 2** Kaplan–Meier curves of the relapse-free interval in children with P-170-positive *vs* P-170-negative ALL.

on the basis of the expression of P-170 and GST-π. Figure 4 shows that the prognosis of the patients according to the probability of remaining in first CCR is more unfavourable by combining both proteins. Similar results were obtained for the relapse rate (Table III). Thirteen out of 22 patients (59%) in the group with coexpression of P-170 and GST-π relapsed. In contrast, only seven of the 38 patients (18%) with no evidence of either resistance protein experienced relapses. Among the 14 patients whose blast cells were P-170 positive but GST-π-negative, four (29%) experienced relapses; among the 30 patients with GST-π-positive and P-170-negative blast cells 14 (47%) developed relapses.

We further analysed the prognostic impact of P-170 and GST-π on the time to relapse in the presence of clinical prognostic factors by multivariate regression methods (Cox regression model). The full model including age, sex, FAB type, immunological subtype, initial peripheral cell count (PBC) as well as P-170 and GST-π revealed a highly significant influence of PBC ($P = 0.001$) and a borderline significant influence of P-170 ($P = 0.08$), whereas all other factors including GST-π were of no significant influence ($P > 0.10$). An analysis of the reduced model with only PBC and P-170 as covariates resulted in a significant influence of both PBC ($P = 0.0001$) and P-170 ($P = 0.025$). The independent effect of P-170 on disease-free survival (in addition to PBC) could be confirmed by an analysis of deviance (deviance = − 2 [log likelihood (PBC) − log likelihood (PBC, P-170)] = − 2 [(− 164.17) − (− 161.91)] = 4.52, $P < 0.05$).

P-170 and GST-π were not totally independent of each other (value of the chi-square test for independency = 2.72, $P = 0.10$). Therefore we checked for a possible prognostic influence of GST-π in addition to that of PBC and P-170 as well as when replacing P-170 by GST-π. Both analyses resulted in no significant additional influence of GST-π ($P = > 0.05$).

### Discussion

Expression of the multidrug resistance gene (*MDR-1*) or its gene product (P-170) has been found in many human solid

**Figure 3** Kaplan–Meier curves of the relapse-free interval in children with GST-π-positive *vs* negative ALL.

**Figure 4** Kaplan–Meier curves of the relapse-free interval in children with (1) P-170-negative/GST-π-negative ($n = 38$), (2) P-170-positive/GST-π-negative ($n = 14$), (3) P-170-negative/GST-π-positive ($n = 30$) and (4) P-170-positive/GST-π-positive ALL ($n = 22$).

tumours (Chan *et al.*, 1990; Efferth *et al.*, 1992; Volm *et al.*, 1992*b*) and in leukaemias (Pirker *et al.*, 1991; Campos *et al.*, 1992). Most studies of leukaemias were performed with acute non-lymphoblastic leukaemia (ANLL) (Holmes *et al.*, 1989; Sato *et al.*, 1990), whereas only few reports on acute lymphoblastic leukaemia (ALL) exist (Rothenberg *et al.*, 1989; Pieters *et al.*, 1992; Goasguen *et al.*, 1993). Using immunohistochemical methods (Musto *et al.*, 1990) or immunoblot techniques (Kuwazuru *et al.*, 1990) P-170 expression was found in six out of 12 and in four out of 11 adult ALL patients respectively. In addition, increased *MDR-1* mRNA transcripts were found in five of 14 adults with ALL (Gruber *et al.*, 1992). In a recent work Goasguen *et al.* (1993) found P-170 expression in 12 out of 36 children (33%) with newly diagnosed ALL. In contrast to these results, Rothenberg *et al.* (1989) found an expression of P-170 in only one out of nine adult ALL patients. Using the monoclonal antibody MRK-16 no expression of P-170 was found in 13 ALL cases (Ito *et al.*, 1989). Pieters *et al.* (1992) analysed cells from 28 untreated ALL patients and found that all samples were P-170 negative. In addition, Ubezio *et al.* (1989) found no increased *MDR-1* mRNA levels in resistant childhood ALL.

**Table III** Relapse rates with respect to P-170 and GST-π coexpression

| | *Patients* n | *Relapse* n *(%)* |
|---|---|---|
| P-170 negative/GST-π negative | 38 | 7 (18) |
| P-170 positive/GST-π negative | 14 | 4 (29) |
| P-170 negative/GST-π positive | 30 | 14 (47) |
| P-170 positive/GST-π positive | 22 | 13 (59) |

These conflicting data were the reason why we decided to determine P-170 in a retrospective study with 104 children with newly diagnosed ALL. In our study we found expression of P-170 in 36 out of 104 samples (35%). One reason for the different results may be the use of different immunohistochemical evaluation criteria. In agreement with other investigators (Musto *et al.*, 1990; Goasguen *et al.*, 1993) the patients in our study were considered to be positive for P-170 if at least 1% of the leukaemic cells were stained. In our opinion, residual disease can also be established by such small cell proportions. For this reason we used this cut-off point for positivity.

We found similar significant results using a cut-off point of 5% or 10% P-170-positive cells (data not shown).

We also compared the results of P-170 with the clinical outcome. As response criteria we used the relapse rate and the relapse-free interval. Both parameters reflect the responsiveness to the applied therapy. We found a significantly lower probability of remaining in first continuous complete remission (CCR) ($P = 0.033$) and a tendency for an increased relapse rate in patients with P-170-positive blast cells ($P = 0.099$). Similar results were published on adult ALL on the basis of *MDR-1* mRNA detection (Marie *et al.*, 1991; Gruber *et al.*, 1992). Musto *et al.* (1991) reported a high risk of early relapse in leukaemia patients with detectable P-170-positive cells in complete remission. Our results may be explained by a selective survival of P-170 cells during the primary therapy. These surviving cells may lead to a minimal residual disease with an increased risk of recurrent disease. The data indicate that the expression of P-170 is a prognostic factor for both relapse rate and relapse-free interval of patients with ALL in addition to clinical prognostic factors.

Several studies suggest that refractoriness to chemotherapy is only partly caused by P-170 and that other resistance mechanisms are also involved in this process (Moscow *et al.*, 1989; Tiedefelt *et al.*, 1992; Volm *et al.*, 1992*a*). Therefore, we also analysed the expression of GST-π in the same patients. We found increased GST-π levels in the blast cells from 52 of 104 patients (50%). Because of its physiological function GST-π expression was found in all samples at a low level so that only patients with moderate or intensive staining were considered to be positive for GST-π overexpression. Gekeler *et al.* (1992) reported moderate GST levels in patients with the initial stage of ALL and with relapsed ALL at the mRNA levels when compared with the cell line CCRF-CEM. Results obtained on initial stage of non-Hodgkin's lymphoma (NHL) (Rodriguez *et al.*, 1993) and relapsed stage of NHL (Cheng *et al.*, 1993) also suggest an involvement of increased GST-π levels in drug resistance of lymphatic tumours. Increased levels of GST-π mRNA have also been observed in untreated adult leukaemias (McQuaid *et al.*, 1989). Tidefelt *et al.* (1992) found that 23 of 59 patients with ANLL were positive for GST-π overexpression using the same discriminating criteria for GST-π estimation as in our study. In addition, in 95% of patients with chronic lymphocytic leukaemia (CLL), Schisselbauer *et al.* (1990) found detectable GST-π levels and a quantitatively increased GST activity in chlorambucil-resistant CLL patients. To our knowledge the present study is the first investigation into the prognostic importance of GST-π in childhood ALL. Our data show an association between increased GST-π levels and a higher relapse rate ($P = 0.003$) as well as a lower probability of the first CCR ($P = 0.01$).

Furthermore, our study suggests a tendency for simultaneous expression of P-170 and GST-π ($P = 0.099$) and the two parameters are probably not independent of each other. Similar results were obtained in NHL (Cheng *et al.*, 1993) and in lung tumours (Volm *et al.*, 1991, 1992*a*). In contrast to this, no correlation was found between the parameters in primary NHL (Rodriguez *et al.*, 1993). Holmes *et al.* (1990) observed GST-π overexpression independent of *MDR-1* gene expression. By combing both parameters in our study, the prognostic significance with respect to the relapse-free interval and the relapse rate was improved. In a univariate analysis we showed that P-170- and GST-π overexpression were independent of sex, FAB type, immunological subtype and the peripheral blast cell count. Goasguen *et al.* (1993) observed a significantly higher expression of P-170 in B-type ALL *vs* precursor B-ALL. We investigated six patients with B-ALL and found four to be positive for P-170 (unpublished data). In fact, the staining in these cells was more intensive and the proportion of positive cells was higher than in non-B-ALL. B-ALL indeed is known to have different biological and clinical features when compared with non-B-ALL, and so these patients are treated differently. This was the reason why we excluded B-ALL patients from our study.

Although we observed a significant prognostic power of GST-π in the univariate analysis ($P = 0.032$), this could not be confirmed in the multivariate analysis when PBC and P-170 were included. The effect of GST-π seems partly to be due to its minor correlation ($P = 0.10$) with P-170 and possibly higher order effects which have been beyond the aim of this investigation. Thus, our results suggest that only P-glycoprotein has prognostic significance in ALL. In earlier investigations we found that no correlation exists between the expression of dihydrofolate reductase, thymidylate synthase, DNA topoisomerase II or metallothionein and the clinical outcome of children with ALL (Sauerbrey *et al.*, 1994; Stammler *et al.*, 1994; Volm *et al.*, 1994*a*).

Since drug resistance remains the major obstacle in the treatment of the lymphoblastic leukaemia, interest has also focused on the elucidation of molecular mechanisms of resistance. Although our knowledge as to which factors are responsible for a regulated coexpression of resistance mechanisms is limited, one possibility is that the resistance factors belong to a set of genes which is controlled by general regulatory mechanisms. Indeed, the c-*fos*/c-*jun* protein complex which binds specifically to AP-1 affects the transcriptional expression of several cellular genes. Interestingly, P-glycoprotein and glutathione *S*-transferase contain an AP-1 motif, thus these genes may also be regulated by the cellular oncogenes c-*fos* and c-*jun* (Angel & Karin, 1991; Teeter *et al.*, 1991). Indeed, in earlier studies (Volm *et al.*, 1994*b*) we found that the relapse-free intervals were lower in patients with Fos- and Jun-positive leukaemic cells than in patients with Fos- and Jun-negative tumour cells. Additionally, nuclear protein kinase C is of high functional importance as a stimulator of the activity of proto-oncogenes such as c-*fos* and c-*jun*. Earlier results of univariate and multivariate analyses demonstrated that in addition to the clinical prognostic indicators protein kinase C is a significant prognostic factor for relapse rate, relapse-free intervals and overall survival times in ALL of children (Volm *et al.*, 1994*c*).

For the statistical analysis we are grateful to Dr L. Edler, Department of Biostatistics, German Cancer Research Center, Heidelberg. We would like to thank Dr R. Häfer for the preparation and the immunophenotyping of the blast cells, H. Malke and M. Reimann for the preparation of the clinical data, H. Grage for his excellent technical assistance and Dr E.W. Pommerenke for critical reading of the manuscript.

### References

ANGEL, P. & KARIN, M. (1991). The role of Jun, Fos, and the AP-1 complex in cell proliferation and transformation. *Biochim. Biophys. Acta*, **1072**, 129–157.

BENNET, J.M., CATOWSKY, D., DANIEL, M.T., FLANDRIN, G., GALTON, G.A.R., GRALNICK, H.R. & SULTAN, C. (1976). Proposal for the classification of the acute leukemias. French American British (FAB) cooperative group. *Br. J. Haematol.*, **33**, 451–458.

BLACK, S.M. & WOLF, C.R. (1991). The role of glutathione-dependent enzymes in drug resistance. *Pharmacol. Ther.*, **51**, 139–154.

BYAR, D.P. (1982). Analysis of survival data: Cox and Weibull models with covariates. In *Statistics in Medical Research. Methods and Issues with Application in Cancer Research*, Mike, V. & Stanley, K.E. (eds) pp. 365–401. John Wiley: New York.

CAMPOS, L., GUYOTAT, D., ARCHIMBAUD, E., CALMARD-ORIOL, P., TSURUO, T., TRONCY, J., TRAILLE, D. & FIERE, D. (1992). Clinical significance of multidrug resistance P-glycoprotein expression on acute nonlymphoblastic leukemia cells at diagnosis. *Blood*, **79**, 473–476.

CHAN, H.S.L., THORNER, P.S., HADDAD, G. & LING, V. (1990). Immunohistochemical detection of P-glycoprotein: prognostic correlation in soft tissue sarcoma of childhood. *J. Clin. Oncol.*, **8**, 689–704.

CHENG, A.L., SU, I.J., CHEN, Y.C., LEE, T.C. & WANG, C.H. (1993). Expression of P-glycoprotein and glutathione-S-transferase in recurrent lymphomas: the possible role of Epstein–Barr virus, immunophenotypes, and other predisposing factors. *J. Clin. Oncol.*, **11**, 109–115.

COX, D.R. (1972). Regression models and life tables. *J. R. Stat. Soc. (B)*, **34**, 187–220.

ENDICOTT, J.A. & LING, V. (1989). The biochemistry of P-glycoprotein-mediated multidrug resistance. *Annu. Rev. Biochem.*, **58**, 137–171.

EFFERTH, T., MATTERN, J. & VOLM, M. (1992). Immunohistochemical detection of P-glycoprotein, glutathione-S-transferase and DNA topoisomerase II in human tumors. *Oncology*, **49**, 368–375.

GEKELER, V., FRESE, G., NOLLER, A., HANDGRETINGER, R., WILISCH, A., SCHMIDT, H., MULLER, C.P., DOPFER, R., KLINGEBIEL, T., DIDDENS, H., PROBST, H. & NIETHAMMER, D. (1992). MDR1/P-glycoprotein, topoisomerase and glutathione-S-transferase π gene expression in primary and relapsed state adult and childhood leukemias. *Br. J. Cancer*, **66**, 507–517.

GOASGUEN, J.E., DOSSOT, J.M., FARDEL, O., LEMEE, F., LEGALL, E., LEBLAY, R., LEPRISE, P.Y., CHAPERON, J. & FAUCHOT, R. (1993). Expression of the multidrug resistance-associated P-glycoprotein (P-170) in 59 cases of de novo acute lymphoblastic leukemia: prognostic implications. *Blood*, **81**, 2394–2398.

GRUBER, A., VITOLS, S., NORGREN, S., ARESTRÖM, I., PETERSON, C., BJÖRKHOLM, M., REIZENSTEIN, P. & LUTHMAN, H. (1992). Quantitative determination of mdr1 gene expression in leukemic cells from patients with acute leukemia. *Br. J. Cancer*, **66**, 266–272.

HOLMES, J., JACOBS, A., CARTER, G., JANOWSKA-WIECZOREK, A. & PADUA, R.A. (1989). Multidrug resistance in haemopoietic cell lines, myelodysblastic syndromes and acute myeloblastic leukemia. *Br. J. Haematol.*, **72**, 40–44.

HOLMES, J., WAREING, C., JACOBS, A., HAYES, D., PADUA, R.A. & WOLF, C.R. (1990). Glutathione-S-transferase pi expression in leukemia: a comparative analysis with mdr-1 data. *Br. J. Cancer*, **62**, 209–212.

ITO, Y., TANIMOTO, M., KUMAZAWA, T., OKUMURA, M., MORISHIMA, Y., OHNO, R. & SAITO, H. (1989). Increased P-glycoprotein expression and multidrug-resistant gene (mdr1) amplification are infrequently found in fresh acute leukemia cells. *Cancer*, **63**, 1534–1538.

KAPLAN, E.L. & MEIER, P. (1956). Nonparametric estimation from incomplete observations. *J. Am. Stat. Assoc.*, **53**, 457–481.

KUWAZURU, Y., YOSHIMURA, A., HANADA, S., UTSUNOMIYA, A., MAKINO, T., ISHIBASHI, K., KODAMA, M., IWAHASHI, M., ARIMA, T. & AKIYAMA, S.I. (1990). Expression of the multidrug transporter P-glycoprotein, in acute leukemia cells and correlation to clinical drug resistance. *Cancer*, **66**, 868–873

MCQUAID, S., MCCANN, S., DALY, P., LAWLOR, E. & HUMPHRIES, P. (1989). Observations on the transcriptional activity of the gluthathione-S-transferase π gene in human haematological malignancies and in the peripheral leucocytes of cancer patients under chemotherapy. *Br. J. Cancer*, **59**, 540–543.

MARIE, J.P., ZITTOUN, R. & SIKIC, B.I. (1991). Multidrug resistance (mdr1) gene expression in adult acute leukemias: correlation with treatment outcome and in vitro drug sensitivity. *Blood*, **78**, 586–592.

MOSCOW, J.A., FAIRCHILD, C.R., MADDEN, M.J., RANSOM, D.T., WIEAND, H.S., O'BRIEN, E.E., POPLACK, D.G., COSSMAN, J., MYERS, C.E. & COWAN, K.H. (1989). Expression of anionic glutathione-S-transferase and P-glycoprotein genes in human tissues and tumors. *Cancer Res.*, **49**, 1422–1428.

MUSTO, P., CASCAVILLA, N., DI RENZO, N., LADOGANA, S., LA SALA, A., MELILLO, L., NOBILE, M., MATERA, R., LOMBARDI, G. & CAROTENUTO, M. (1990). Clinical relevance of immunocytochemical detection of multidrug resistance-associated P-glycoprotein in hematologic malignancies. *Tumori*, **76**, 353–359.

MUSTO, P., MELILLO, L., LOMBARDI, G., MATERA, R., DIGIORGIO, G. & COROTENUTO, M. (1991). High risk of early relapse for leukemia patients with presence of multidrug resistance associated with P-glycoprotein-positive cells in complete remission. *Br. J. Haematol.*, **77**, 50–53.

PIETERS, R., HONGO, T., LOONEN, A.H., HUISMAN, D.R., BROXTERMAN, H.J., HÄHLEN, K. & VEERMAN, A.J.P. (1992). Different types of non-P-glycoprotein-mediated multiple drug resistance in children with relapsed acute lymphoblastic leukemia. *Br. J. Cancer*, **65**, 691–697.

PIRKER, R., WALLNER, J., GEISSLER, K., LINKESCH, W., HAAS, O.A., BETTELHEIM, P., HOPFNER, M., SCHERRER, R., VALENT, P., HAVELEC, L., LUDWIG, H. & LECHNER, K. (1991). MDR1 gene expression and treatment outcome in acute myeloid leukemia. *J. Natl Cancer Inst.*, **83**, 708–712.

RIEHM, H., EBELL, W., FEICKERT, H.J. & REITER, A. (1992). Acute lymphoblastic leukemia. In *Cancer in Children: Clinical Management*, Voute, P.A., Barrett, A., Lemerle, J. (eds) pp. 85–106. Springer: Berlin.

RODRIGUEZ, C., COMMES, T., ROBERT, J. & ROSSI, J.F. (1993). Expression of P-glycoprotein and anionic glutathione S-transferase genes in non-Hodgkin lymphoma. *Leukemia Res.*, **17**, 149–156.

ROTHENBERG, M.L., MICKLEY, L.A., COLE, D.E., BALIS, F.M., TSURUO, T., POPLACK, D.G. & FOJO, A.T. (1989). Expression of the mdr-1/P-170 gene in patients with acute lymphoblastic leukemia. *Blood*, **74**, 1388–1395.

SATO, H., PREISLER, H., DAY, R., RAZA, A., LARSON, R., BROWMAN, G., GOLDBERG, J., VOGLER, R., GRUNWALD, H., GOTTLIEB, A., BENNET, J., GOTTESMAN, M. & PASTAN, I. (1990). MDR1 transcript levels as an indication of resistant disease in acute myelogenous leukemia. *Br. J. Haematol.*, **75**, 340–345.

SAUERBREY, A., ZINTL, F. & VOLM, M. (1994). Expression of metallothionein in initial and relapsed childhood acute lymphoblastic leukemia. *Ann. Hematol.*, (in press).

SCHISSELBAUER, J.C., SILBER, R., PAPADOPOULOS, E., ABRAMS, K., LACRETA, F.P. & TEW, K.D. (1990). Characterization of glutathione S-transferase expression in lymphocytes from chronic lymphocytic leukemia patients. *Cancer Res.*, **50**, 3562–3568.

SCHLAIFER, D., LAURENT, G., CHITTAL, S., TSURUO, T., SOUES, S., MULLER, G., CHARCOSSET, J.Y., ALARD, C., BROUSSET, P., MAZERROLLES, C. & DELDOL, G. (1990). Immunohistochemical detection of multidrug-resistance associated P-glycoprotein in tumour and stromal cells of human cancers. *Br. J. Cancer*, **62**, 177–182.

STAMMLER, G., SAUERBREY, A. & VOLM, M. (1994). Determination of DNA topoisomerase II in newly diagnosed childhood acute lymphoblastic leukemia by immunocytochemistry and RT-PCR. *Cancer Lett.* (in press).

TEETER, L.D., ECKERSBERG, T., TSAI, Y. & KUO, M.T. (1991). Analysis of the chinese hamster P-glycoprotein (multidrug resistance gen pgp1 reveals that the AP-1 site is essential for full promoter activity. *Cell Growth Different.*, **2**, 429–437.

TIDEFELT, U., ELMHORN-ROSENBORG, A., PAUL, C., HAO, X.Y., MANNERVIK, B. & ERIKSSON, L.C. (1992). Expression of glutathione transferase $\pi$ as a predictor for treatment results at different stages of acute nonlymphoblastic leukemia. *Cancer Res.*, **52**, 3281–3285.

UBEZIO, P., LIMONTA, M., D'INCALCI, M., DAMIA, G., MASERA, G., GIUDICI, G., WOLFERTON, J.S. & BECK, W.T. (1989). Failure to detect the P-glycoprotein multidrug resistant phenotype in cases of resistant childhood acute lymphocytic leukemia. *Eur. J. Cancer Clin. Oncol.*, **25**, 1895–1897.

VOLM, M., BAK, M., EFFERTH, T., LATHAN, B. & MATTERN, J. (1988). Immunocytochemical detection of resistance-associated glycoprotein in tissue culture cells, ascites tumors and human xenografts by Mab 265/F4. *Anticancer Res.*, **8**, 531–536.

VOLM, M., MATTERN, J. & SAMSEL, B. (1991). Overexpression of P-glycoprotein and glutathione-S-transferase-$\pi$ in resistant non-small cell carcinomas of smokers. *Br. J. Cancer*, **64**, 700–704.

VOLM, M., MATTERN, J. & SAMSEL, B. (1992*a*). Relationship of inherent resistance to doxorubicin, proliferative activity and expression of P-glycoprotein 170, and glutathione-S-transferase $\pi$ in human lung tumors. *Cancer*, **70**, 764–769.

VOLM, M., MATTERN, J. EFFERTH, T. & W.POMMERENKE, E. (1992*b*). Expression of several resistance mecahnisms in untreated human kidney and lung carcinomas. *Anticancer Res.*, **12**, 1063–1068.

VOLM, M., SAUERBREY, A. & ZINTL, F. (1994*a*). Dihydrofolate-reductase and thymidylate-synthase in childhood acute lymphoblastic leukemia. *Anticancer Res.*, **14**, 1377–1382.

VOLM, M., SAUERBREY, A., STAMMLER, G. & ZINTL, F. (1994*b*). Detection of Fos, Jun and RAS in newly diagnosed childhood acute lymphoblastic leukemia by immunocytochemistry and PCR. *Int. J. Oncol.*, **4**, 1251–1256.

VOLM, M., SAUERBREY, A. & ZINTL, F. (1994*c*). Prognostic significance of protein kinase C in newly diagnosed childhood acute lymphoblastic leukemia. *Int. J. Oncol.*, **4**, 363–368.

ZINTL, F., MALKE, H., REIMANN, M., DOMULA, M., DÖRFFEL, W., EGGERS, G., EXADAKTYLOS, P., HILGENFELD, E., KOTTE, W., KRAUSE, I., KUNERT, W., MITTLER, U., MÖBIUS, D., REDDEMANN, H., WEINMANN, G. & WEIßBACH, G. (1993). Results with randomized BFM adopted studies for ALL therapy in childhood in East German countries. In *Acute Leukemias IV: Prognostic Factors*, Büchner, T., Schellong, G., Hiddemann, W., Urbanitz, D. & Ritter, J. (eds) pp. 179–186. Springer: Berlin.